# High SNR Φ-OTDR with Multi-Transverse Modes Heterodyne Matched-Filtering Technology

**DOI:** 10.3390/s21227460

**Published:** 2021-11-10

**Authors:** Yifan Liu, Junqi Yang, Bingyan Wu, Bin Lu, Luwei Shuai, Zhaoyong Wang, Lei Ye, Kang Ying, Qing Ye, Ronghui Qu, Haiwen Cai

**Affiliations:** 1 Key Laboratory of Space Laser Communication and Detection Technology, Shanghai Institute of Optics and Fine Mechanics, Chinese Academy of Sciences, Shanghai 201800, China; liuyifan@siom.ac.cn (Y.L.); jqyang@siom.ac.cn (J.Y.); bywu@siom.ac.cn (B.W.); lubin@siom.ac.cn (B.L.); shuailuwei@siom.ac.cn (L.S.); wzhy0101@siom.ac.cn (Z.W.); yelei@siom.ac.cn (L.Y.); yingk0917@siom.ac.cn (K.Y.); yeqing@siom.ac.cn (Q.Y.); rhqu@siom.ac.cn (R.Q.); 2 Center of Materials Science and Optoelectronics Engineering, University of Chinese Academy of Sciences, Beijing 100049, China

**Keywords:** Φ-OTDR, few-mode fiber, multi-transverse modes heterodyne matched-filtering technology, high-SNR

## Abstract

Phase-sensitive optical time domain reflectometer (Φ-OTDR) has attracted attention in scientific research and industry because of its distributed dynamic linear response to external disturbances. However, the signal-to-noise ratio (SNR) of Φ-OTDR is still a limited factor by the weak Rayleigh Backscattering coefficient. Here, the multi-transverse modes heterodyne matched-filtering technology is proposed to improve the system SNR. The capture efficiency and nonlinear threshold are increased with multiple transverse modes in few-mode fibers; the incident light energy is permitted to be enlarged by a wider probe pulse by using heterodyne matched-filtering without spatial resolution being deteriorated. As far as we know, this is the first time that both multi-transverse modes integration method and digital heterodyne matched filtering method have been used to improve the SNR of Φ-OTDR simultaneously. Experimental results show that the noise floor is reduced by 11.4 dB, while the target signal is kept. We believe that this proposed method will help DAS find important applications in marine acoustic detection and seismic detection.

## 1. Introduction

Phase-sensitive optical time domain reflectometer (Φ-OTDR) can detect and locate one or more vibration events occurring at any position along the optical fiber, and has a wide range of applications in boundary intrusion monitoring [1,2], oil and gas pipeline safety monitoring [3] and structural health monitoring [4,5]. The Φ-OTDR scheme was first proposed by H. F. Taylor et al. to achieve intrusion detection in 1993 [6]. Due to the unique distributed dynamic detection characteristics of Φ-OTDR, an increasing number of researchers have been involved in related fields and research. Many schemes have been proposed to improve its performance, such as coherent detection and specific signal processing for long-distance detection [7], phase demodulation for quantitative measurement [8], and multi-frequency sources for detection bandwidth improvement [9]. However, there are still some inherent disadvantages that restrain their practical adoption and use for certain applications, such as seismic detection [10] and marine acoustic detection [11], which need high SNR and sensitivity. Recently, using optical fiber cable as a distributed sensor to monitor earth dynamics in the ocean is a promising and feasible solution, which gives new opportunities for optical fiber sensing technology for marine application [12]. In conventional Φ-OTDR, the Rayleigh Backscattering (RBS) coefficient is inherently quite low, which will cause larger variance of the phase noise, and the SNR will severely deteriorate [13]. Increasing the input pulse power could help, but the peak power is ultimately limited by the nonlinear effects in the fiber. At the same time, the spatial resolution will limit the pulse width, pulse energy, and thus, SNR. Therefore, the improvement of SNR is a meaningful but difficult challenge.

In order to improve the system SNR, researchers carried out several studies. On the one hand, some fiber processing methods are proposed to increase RBS coefficient, including weak reflection points [14], ultraviolet irradiation [15], ultra-fast laser processing [16], etc. These methods are effective to improve SNR, but not suitable for some cost-conscious applications and mass production. On the other hand, multiple transverse modes are utilized to enlarge the capture efficiency. In 2018, the U.S. Naval Research Laboratory firstly introduced a multi-mode fiber to improve SNR, with high Rayleigh scattering capture efficiency and multiple transverse modes [17]. An off-axis holographic system is constructed for point sensing with a high-speed camera. Recently, Chen Mengmeng et al. combined Rayleigh scattering signals of two different modes in a two-mode fiber to achieve improved SNR of 2.52 dB [18]. At the same time, using space-division multiplexed (SDM) pulse probes in a FMF can suppress interference fading effectively [19]. Apparently, the characteristic of multi-transverse modes is helpful for SNR enhancement. It is believed that the few-mode fiber (FMF) will be a good choice with scarcely any mode coupling.

In this paper, a high SNR Φ-OTDR system with multi-transverse modes heterodyne matched-filtering (MHMF) technology is proposed. Firstly, due to the large core diameter of FMFs, the scattered light can be coupled to multiple permitted modes and transport in the fiber. More RBS light modes will bring more information, and the multi-transverse modes integration (MTMI) method will effectively suppress the noise floor. Secondly, the digital heterodyne matched filtering (DHMF) method will break through the tradeoff between pulse energy and spatial resolution, bring stronger RBS with wider pulses, and improve SNR without sacrificing spatial resolution. The experimental results show that the Φ-OTDR system based on MHMF can improve the SNR by 11.4 dB. This result paves the way for advanced acoustic applications of Φ-OTDR, such as acoustic emission and marine acoustics.

## 2. Principle

### 2.1. Chirping Laser Source and Multi-Transverse Modes Excitation

**Figure 1:**
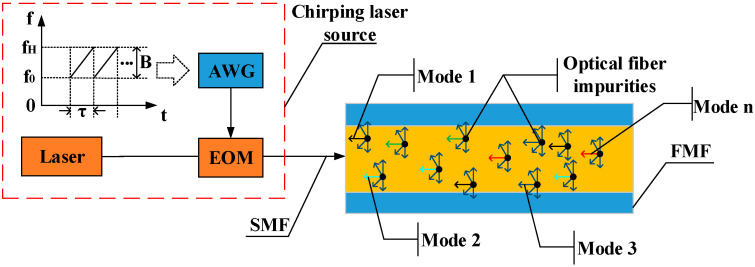
Schematic diagram of chirping laser source for MHMF.

The schematic diagram of the chirping laser source for MHMF is shown in Figure 1. The chirping laser source consists of a narrow linewidth laser and an electro-optic modulator (EOM). EOM is modulated by an arbitrary waveform generator (AWG), with a linear frequency modulated (LFM) sequence. The chirping laser source can be expressed as

(1) $$E=E_{0}exp\left(j\omega_{c}t+j2\pi f_{0}t+j\pi Kt^{2}\right),$$

where $E_{0}$ is the amplitude of the incident electric field, $\omega_{c}$ is the angular frequency of the light wave, $f_{0}$ and K=B/τ are the initial frequency and sweep slope of the LFM signal, respectively, τ is the sweep period, and B is the sweep range of the LFM signal.

When the light beams emitted by the chirping laser source enter a FMF, light beams are scattered in all directions, parts of which leak into the cladding, and only a small part can be captured by the fiber core and propagated backwards. Due to the large core diameter of a FMF, the scattered light can be coupled to all the permitted transverse modes in the fiber. Therefore, more RBS light will be captured in the core for transmission. The RBS signal from a FMF can be expressed as

(2) $$E_{B}=\overset{N}{\underset{n=1}{\sum }}\left|{\hat{r}}_{Ray}\right|E_{m}e^{-\alpha z}B_{mn}exp\left(j\phi_{z,n}+j\phi_{Ray}\right),$$

where m represents the transmission transverse mode at the signal injection end. As the quasi-single mode (QSM) operated FMF offers higher SNR than that of other degenerate higher order modes injection [20], the QSM-operated-FMF (m=1) is chosen as the single-transverse mode (STM) injection in this work. n=1,2,3,⋯,N represents the scattering transverse mode at the signal receiving end. $B_{mn}$ is the RBS capture fraction, which is used to represent the ratio of scattering power into the $n^{th}$ transverse mode to the total scattering power. α is the fiber attenuation coefficient. $\left|{\hat{r}}_{Ray}\right|$ is the amplitude of the synthetic Rayleigh scattering coefficient. $\phi_{Ray}$ is the Rayleigh coherent phase. $\phi_{z,n}$ is the transmission phase, which is mainly related to external disturbances.

Diversity reception is adopted at the receiving end, so RBS light of the $n^{th}$ transverse mode can be expressed as

(3) $$E_{B,n}=\left|{\hat{r}}_{Ray}\right|E_{1}e^{-\alpha z}B_{1n}exp\left(j\phi_{z,n}+j\phi_{Ray}\right),$$

### 2.2. Digital Heterodyne Matched Filtering

According to the principle of heterodyne detection, RBS is mixed with the local oscillator reference light $E_{L}=E_{L0}e^{j\omega_{L}t}$ by a balanced coupler. The response equations of the balanced coupler are as follows:

(4) $$\frac{E_{h}=E_{L}+jE_{B,n},}{E_{c}=jE_{L}+E_{B,n},}$$

The output signal can be expressed as

(5) $$\frac{I\left(t\right)=A_{n}exp\left(j2\pi f_{0}t+j\pi Kt^{2}\right),}{A_{n}=2E_{0}E_{L0}\left|{\hat{r}}_{Ray}\right|e^{-\alpha z}B_{1n}exp\left(j\phi_{z,n}+j\phi_{Ray}\right),}$$

In order to obtain external disturbances from the chirping output signals, a matched filter is designed in the digital domain. For the signal detected in Equation (5), the matched filter is expressed as [21,22]

(6) $$h\left(t\right)=exp\left(j2\pi f_{0}t-j\pi Kt^{2}\right),$$

**Figure 2:**
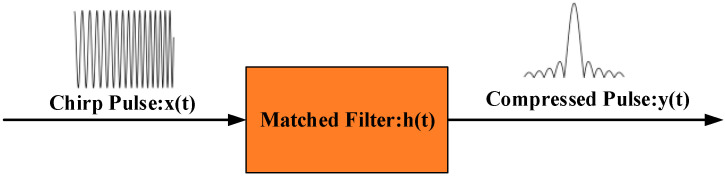
The schematic diagram of DHMF [23].

The DHMF principle is shown in Figure 2. The signal after DHMF is obtained as

(7) $$y\left(t\right)=I\left(t\right)*h\left(t\right)=A_{n}\frac{sin\left[\pi KT\left(1-\frac{\left|t\right|}{T}\right)t\right]}{\pi Kt}exp\left(j2\pi f_{0}t\right),$$

where T is the probe pulse width. Thus, the chirping carrier is eliminated, and external disturbances can be demodulated by digital coherent phase demodulation [8]. More significantly, the spatial resolution becomes as follows:

(8) $$R=\frac{c}{2nKT}=\frac{c}{2nB},$$

which is determined by B rather than T. That is, MHMF can boost the pulse energy by increasing the pulse width, while the spatial resolution is kept. Moreover, the threshold of nonlinear effect will also increase, due to the wider frequency linewidth of probe pulses. To some extent, SNR is determined by pulse and RBS energy. Thus, MHMF is beneficial for RBS energy and SNR improvement.

### 2.3. Multi-Transverse Modes Integration

With I/Q signal demodulation [8], the output vector can be simplified as

(9) $${\hat{P}}_{1n}=2E_{0}E_{L0}\left|{\hat{r}}_{Ray}\right|e^{-\alpha z}B_{1n}exp\left(j\phi_{z,n}+j\phi_{Ray}\right)\cdot \frac{sin\left[\pi KT\left(1-\frac{\left|t\right|}{T}\right)t\right]}{\pi Kt}=A_{1n}exp\left(j\phi_{1n}\right),$$

Here, $A_{1n}$ and $\phi_{1n}$ are the amplitude term and the phase term, respectively. Then the rotation vector method [24] is used to compensate the initial phase, at which time the vector form of the signal can be expressed as

(10) $${\hat{P}}_{1n}^{,}=\frac{{\hat{P}}_{1n}\cdot {\hat{P}}_{1n}^{*}}{\left|{\hat{P}}_{1n}\right|}=A_{1n}exp\left(j\varphi_{1n}\right),$$

Further, the signals of multi-transverse modes are integrated. In order to reduce the influence of the phase error when fading occurs on the quality of integration results, we set signal amplitude $A_{1n}^{\prime }$ as the weight information to measure its phase quality in the integrating process. At the same time, we use spatial shift-difference method to eliminate the cumulative phase difference with distance between each transverse mode [25]. Then the signal after MTMI can be expressed as

(11) $$\hat{P}=\overset{N}{\underset{n=1}{\sum }}A_{1n}^{\prime }exp\left(j\varphi_{1n}^{\prime }\right),$$

where $A_{1n}^{\prime }$ is the smaller amplitude of two differential points $z_{1}=z-dz/2$ and $z_{2}=z+dz/2$, $\varphi_{1n}^{\prime }$ is the differential phase of two differential points $z_{1}$ and $z_{2}$.

MTMI can effectively integrate signals of multiple transverse modes. When the probe pulse is launched into the fundamental transverse mode of a photon lantern (MMUX), the RBS light can couple all the transverse modes in the fiber. Then the signals are received by the photon lantern (DEMUX) and integrated by MTMI method. After integrating signals of N transverse modes, the SNR can be described as follows:

(12) $$SNR^{\prime }\left(z\right)=\frac{{\sigma_{\phi }^{\prime }}^{2}}{{\sigma_{N}^{\prime }}^{2}\left[1/A^{2}\left(z\right)\right]}\approx \frac{\sigma_{\phi }^{2}}{N\sigma_{N}^{2}\cdot {\left\{1/\left[N\cdot A\left(z\right)\right]\right\}}^{2}}=N\cdot SNR\left(z\right),$$

where $\sigma_{\phi }^{2}$ and $\sigma_{N}^{2}$ are the variance of signal and system noise, respectively. In Equation (12), we ignore the different capture efficiency of RBS light in each transverse mode, and consider that each transverse mode transmits independently. Therefore, the SNR will improve by 10lgN dB theoretically [26]. Photon lantern provides low-loss interfaces between single-mode and multimode systems [27]. At the same time, it uses mode matching techniques to minimize insertion losses and mode dependent losses, which allow us to ignore its effect on SNR improvement. In addition, we do not consider the effect of mode crosstalk on SNR improvement. The reason for this will be explained in the later section. That is, MHMF can boost the amplitude of signal by increasing RBS light in FMF. To some extent, SNR is determined by the amplitude of the signal. Thus, MTMI can improve SNR effectively.

## 3. Experiment

### 3.1. SNR Improvement Only with MTMI

**Figure 3:**
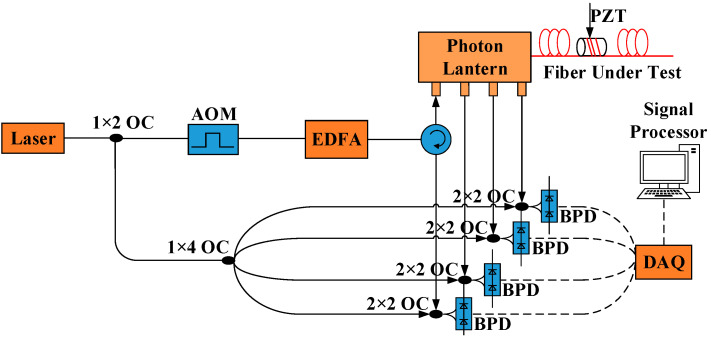
Experimental setup of Φ-OTDR system based on MTMI technology.

Firstly, we verify the SNR improvement with MTMI technology and a single frequency light source. The system setup is shown in Figure 3. A continuous-wave narrow-linewidth laser is split into two paths by a 1 × 2 OC, 90% is used as probe light and 10% is used as local reference light. The local reference light is divided into four reference lights by a 1 × 4 OC. The probe light is chopped into a pulse by an acousto-optic modulator (AOM) with a frequency shift of 160 MHz. The pulse width is 100 ns, the pulse repetition frequency (PRF) is 5 kHz and the corresponding spatial resolution is 10 m. Amplified by an EDFA, the pulse is launched into the fundamental transverse mode port of the photon lantern (MMUX) through a circulator (STM injection), then launched into the fiber under test (FUT). The scattered signals of multi-transverse modes are detected from the corresponding transverse mode ports of the photon lantern (DEMUX), respectively. After mixing with their respective local reference light, RBS of each transverse mode is received by a BPD with bandwidth of 800 MHz. Then the electric signal is sampled by a DAQ with a sampling rate of 500 MS/s. A short segment of FUT is wound around a cylindrical piezoelectric sensor (PZT). The length of FUT is about 2 km, and the PZT was driven with a sinusoidal vibration signal with a frequency of 625 Hz.

In the experiment, the MMUX and the DEMUX are adopted by an all-fiber photon lantern. The transverse mode distribution of it is LP01, LP02, LP11a/b and LP21a/b; The mode extinction of it is >10 dB (LP01, LP11a/b) and >7 dB (LP21a/b, LP02); The mode power ration of the scattered signals is about LP21a/b:LP11a/b:LP02:LP01 = 1:2:2:8; The insertion losses of the photon lantern is about 2.3 dB. We select a graded-index four-mode fiber, which has a small differential group delay and a relatively large mode interval effective refractive index difference. In addition, because of the high selectivity of the photon lantern, we believe that the mode crosstalk has little influence on the SNR improvement.

**Figure 4:**
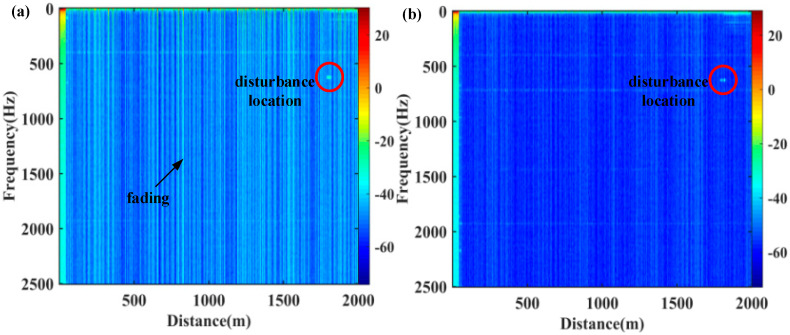
The power spectral density (PSD) of differential phase. (**a**) STM; (**b**) MTMI. The bright lines indicated by the arrow in (**a**) are fading; The bright spots within the circles in (**a**,**b**) are the disturbance locations.

**Figure 5:**
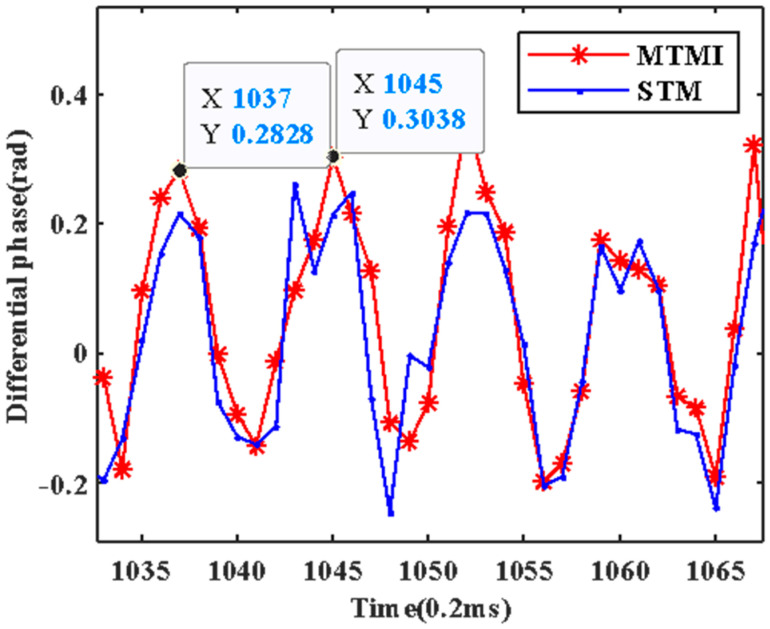
The comparison in the differential phase at the disturbance location between STM and MTMI.

The power spectral density (PSD) of differential phase is calculated, and results of STM and MTMI are shown in Figure 4. It is shown clearly that there are many distortion points from interference fading in the STM result (bright lines). After MTMI, most of the fading disappeared as shown in Figure 4b. At the same time, a disturbance point with a frequency of 625 Hz can be clearly observed. After signal demodulation, the differential phases are shown in Figure 5, and both signals are rebuilt with 8 sample points in a period, which is consistent with the implemented vibration. There are some severe distortions in the STM signal, but the MTMI result is smoother. That is, the system noise is suppressed to a certain extent.

**Figure 6:**
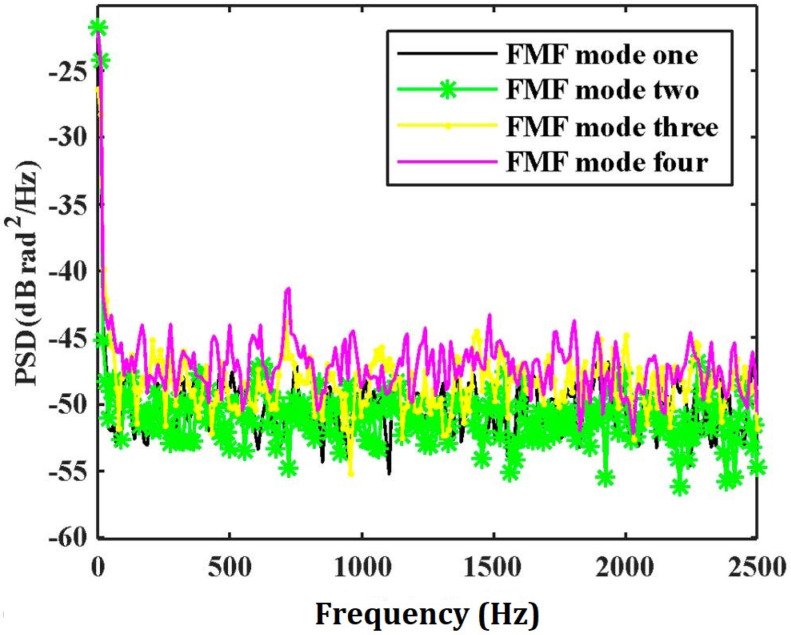
The PSD without disturbance location in each transverse mode in the FMF.

**Figure 7:**
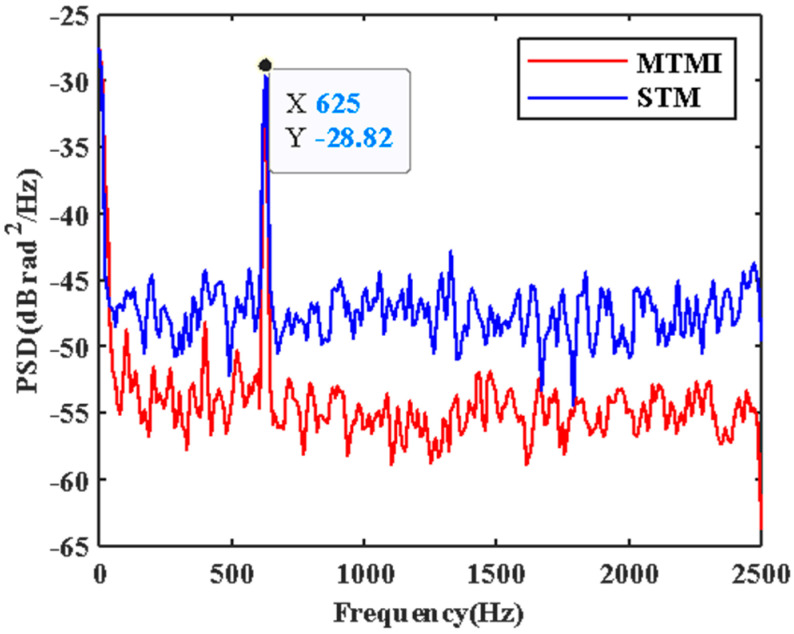
The comparison in the PSD at the disturbance location between STM and MTMI.

**Figure 8:**
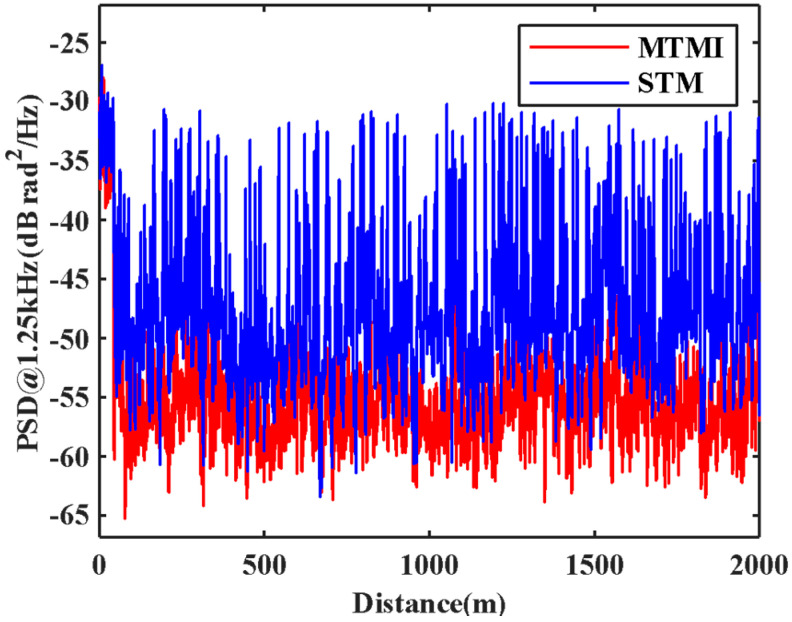
The comparison in the PSD along the distance at the frequency of 1.25kHz between STM and MTMI.

In order to evaluate the SNR improvement of the system quantitatively, the PSD without disturbance in each transverse mode in the FMF is illustrated in Figure 6. We can see the actual noise suppression obtained by each transverse mode is different. We select one of these transverse modes to compare with the result of MTMI, as shown in Figure 7. It can be seen that the frequency of the external disturbance signal is 625 Hz, and the noise floor of the system is reduced. The PSD along the distance at the undisturbed frequency (1.25 kHz) is illustrated in Figure 8. It can be seen that the MTMI does not only eliminate many interference fading points, but also reduces the mean noise floor of the system from −47.8 dB (re 1 rad^2^/Hz) to −55.4 dB. Therefore, the mean noise floor of the system is reduced by 7.6 dB after MTMI. We can see that the experimental value (7.6 dB) is slightly different from the theoretical value (10lg4≈6 dB), because we choose one transverse mode with the worst mean noise floor for comparison.

### 3.2. SNR Improvement with MTMI and DHMF

**Figure 9:**
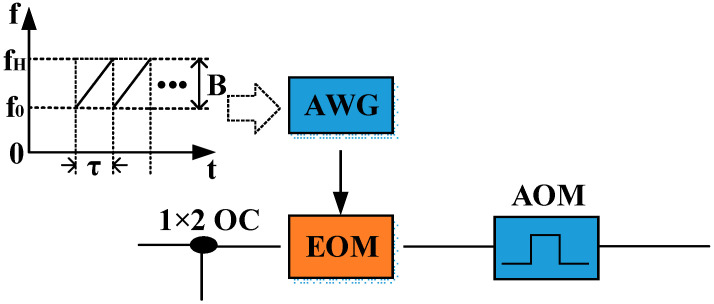
Schematic diagram of generating LFM signal by EOM.

To verify the SNR improvement with MHMF (MTMI and DHMF), the system setup of Figure 3 is introduced and an EOM is added before the AOM. As shown in Figure 9, EOM is modulated by a LFM sequence with the sweep period τ=8 μs and the sweep range B=50 MHz. The system parameters are consistent with Section 3.1, but the pulse width of the AOM is 1.8 μs. The sweep slope of LFM is K=6.25 MHz/μs. The corresponding spatial resolution is about 10 m, which is consistent with the result of Section 3.1.

**Figure 10:**
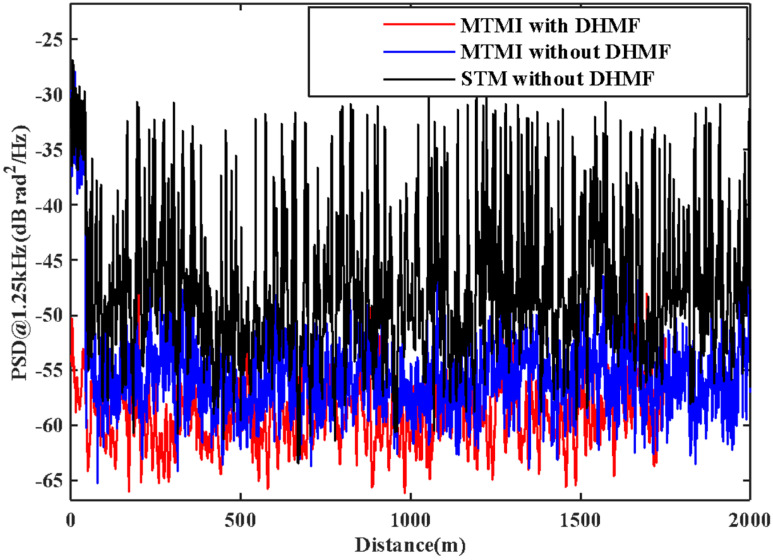
The comparison in the PSD along the distance at the frequency of 1.25 kHz between STM without DHMF, MTMI without DHMF and MTMI with DHMF.

**Figure 11:**
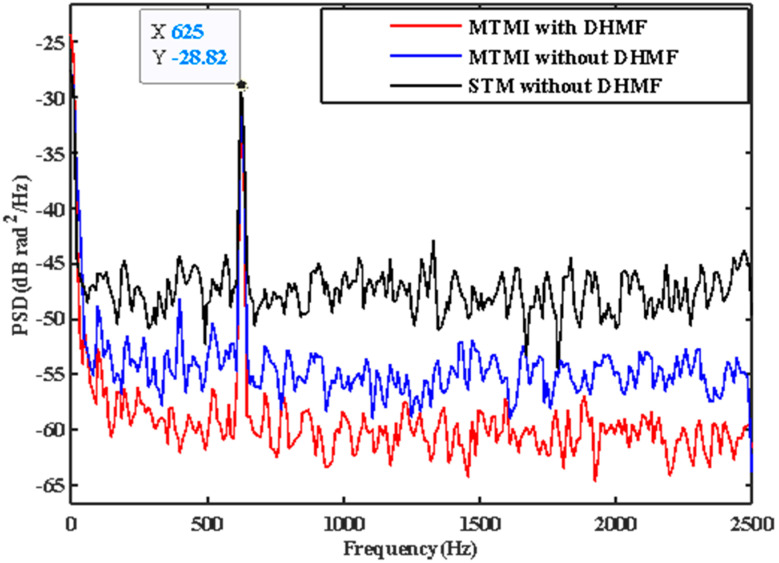
The comparison in the PSD at the disturbance location between STM without DHMF, MTMI without DHMF and MTMI with DHMF.

The collected signals of four transverse modes are analyzed, filtered with DHMF, and demodulated with MTMI. Similarly, in order to evaluate the SNR improvement of the system quantitatively, the PSD along the distance at the undisturbed frequency (1.25 kHz) is illustrated in Figure 10. It can be seen that the MHMF does reduce the mean noise floor of the system to −59.2 dB, which is 3.8 dB lower than that only with the MTMI system. Figure 11 is the PSD at the disturbance location. It can be seen that the frequency of the external disturbance signal is 625 Hz. The mean noise floor of the two groups of experimental results is shown in Table 1. In the second group of experiments, we can see that with DHMF, MTMI can still bring SNR improvement by 7.7 dB, which further verifies the effectiveness of MTMI in improving SNR. At the same time, it can be seen that the mean noise floor of the system is reduced by 11.4 dB after MHMF, while the intensity of the disturbed signal is not significantly reduced.

## 4. Discussion

In MHMF Φ-OTDR scheme, due to the different capture efficiency of RBS light of each scattered mode and the different effective mode field area of each transmission mode, the actual noise suppression obtained by each transverse mode is different, which leads to the difference between the experimental results and the theoretical analysis. At the same time, the mode crosstalk of the photon lantern and the FMF can also affect the experimental results, which are worth further study. Besides, our system is single transverse mode input multiple transverse modes output. We can make full use of the transmission modes in FMFs to achieve multiple transverse modes input multiple transverse modes output. At the same time, DHMF technology is combined to achieve higher SNR.

## 5. Conclusions

In this work, we analyze the factors affecting SNR of the Φ-OTDR system and propose a system with MTMI technology by using the advantages of high capture efficiency, the high nonlinear threshold, and multi-channel independent transmission in FMFs. The preliminary experimental results prove that it is capable not only of effectively suppressing interference fading, but also reducing the mean noise floor of the system by 7.6 dB. Besides, in further experiments, a Φ-OTDR system with MHMF technology is proposed to introduce high energy with a wide pulse. On the basis of MTMI, DHMF technology is added, and experimental results show that compared with the Φ-OTDR system in STM without DHMF, the external disturbance signal can achieve better reconstruction, and the mean noise floor is reduced by 11.4 dB. At the same time, the parameters of a LFM signal can be optimized to achieve a lower noise floor for the system to further meet the requirements of marine acoustic detection, seismic detection and other applications. Furthermore, we can achieve higher SNR by combining larger diversity scales with DHMF technology.

## Figures and Tables

**Table 1:** The mean noise floor of Φ-OTDR system.

Mean Noise Floor	STM	MTMI
without DHMF	−47.8 dB	−55.4 dB
with DHMF	−51.5 dB	−59.2 dB

## Data Availability

Data are contained within the communication.
